# Supplementary material for: Splice-Junction-Based Mapping of Alternative Isoforms in the Human Proteome
Source: Cell Rep. Author manuscript; Available in PMC 2020 Jan 15. (PMC6961840; doi:10.1016/j.celrep.2019.11.026)
Supplement: 2 [file NIHMS1546469-supplement-2.zip › DF1/PXD009737/TestisTrypsin/TestisTrypsin_67_CCDC88B_EGSFAGAAAAPGGAAGEPAGPCPGAAAAEPAGAGA.pdf]

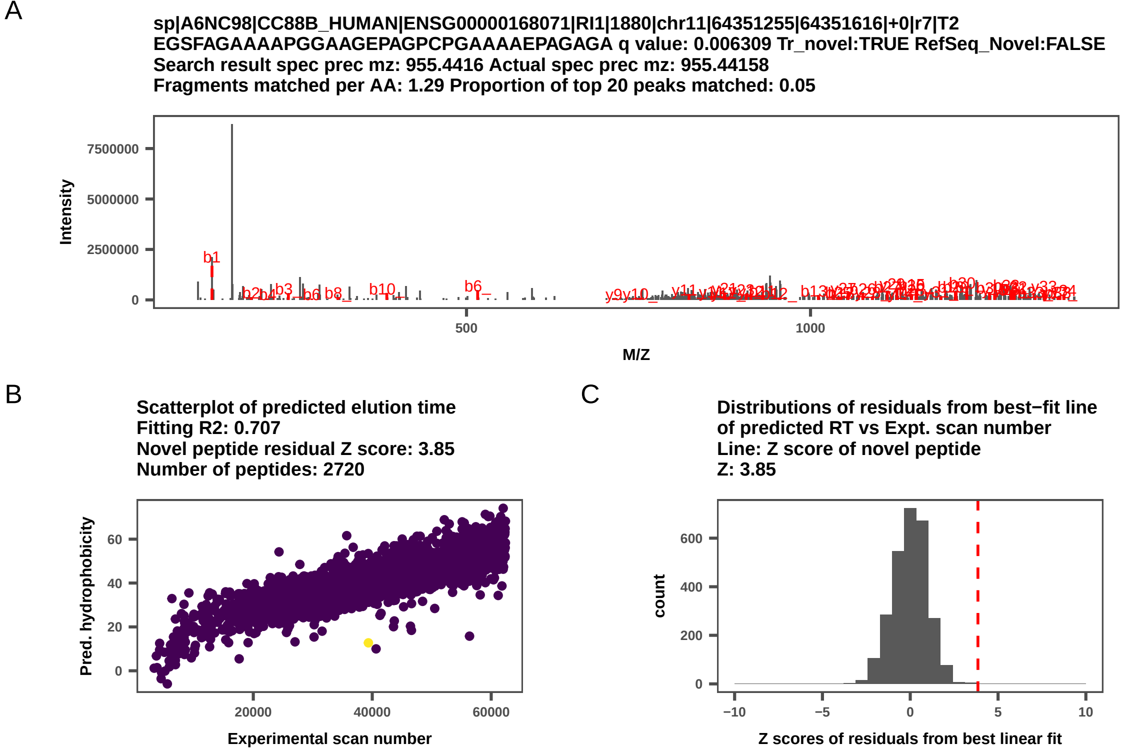
A
sp|A6NC98|CC88B_HUMAN|ENSG00000168071|RI1|1880|chr11|64351255|64351616|+0|r7|T2
EGSFAGAAAAPGGAAGEPAGPCPGAAAAEPAGAGA q value: 0.006309 Tr_novel:TRUE RefSeq_Novel:FALSE
Search result spec prec mz: 955.4416 Actual spec prec mz: 955.44158
Fragments matched per AA: 1.29 Proportion of top 20 peaks matched: 0.05
Intensity
7500000
5000000
2500000
0
b1
b2
b4
b3
b6
b8
b10
b6
y9
y10
y11
b13
b30
500
1000
M/Z
B
Scatterplot of predicted elution time
Fitting R2: 0.707
Novel peptide residual Z score: 3.85
Number of peptides: 2720
Pred. hydrophobicity
60
40
20
0
20000
40000
60000
Experimental scan number
C
Distributions of residuals from best−fit line
of predicted RT vs Expt. scan number
Line: Z score of novel peptide
Z: 3.85
count
600
400
200
0
−10
−5
0
5
10
Z scores of residuals from best linear fit
